# A Novel Endoscopic Anterior Cricoid Rib Grafting: A Feasibility Study in An Animal Model

**DOI:** 10.1002/oto2.70012

**Published:** 2024-09-23

**Authors:** Bshair Aldriweesh, Nasser Almutairi, Waleed Alshareef, Abdullah Sindi, Ahmed Alammar

**Affiliations:** ^1^ Department of Otolaryngology–Head and Neck Surgery King Saud University Medical City, College of Medicine, King Saud University Riyadh Kingdom of Saudi Arabia; ^2^ Present address: Department of Otolaryngology–Head and Neck Surgery Maternity and Children Hospital Makkah Saudi Arabia

**Keywords:** airway stenosis, endoscopic technique, laryngotracheal reconstruction, subglottic stenosis

## Abstract

**Objective:**

The objective of our study was to document the feasibility of a novel endoscopic anterior cricoid split and rib grafting technique in a goat airway model.

**Study Design:**

Feasibility pilot animal study.

**Setting:**

Animal surgical laboratory at a tertiary hospital and research center.

**Methods:**

Three Ardhi goats were utilized. After harvesting and shaping the rib graft, 2 sutures were inserted transversely into the graft. An endoscopic midline anterior cricoid split was performed and extended down through the first tracheal ring, followed by a balloon dilation of the site. Next, the 2 lower and upper graft suture ends were sequentially passed as endo‐extra laryngeal sutures and tied on the anterior neck skin. Laryngeal stent was utilized in 1 goat following graft placement.

**Results:**

The surgery was successful in all included animals and bronchoscopy performed 7 days after surgery, revealed that the anterior graft was in good position. One goat developed surgical site infection leading to partial graft resorption.

**Conclusion:**

This study demonstrated the feasibility of this novel procedure which is potentially useful for patients who are candidates for a single‐stage reconstruction. Future studies should investigate the safety and validity of this technique in a model with subglottic stenosis.

**Level of Evidence:**

NA

Endoscopic airway surgery carries less morbidity and reduces operative time in comparison to open transcervical procedures.^1^ Laryngotracheal expansion surgery encompasses the use of rib graft to augment the airway.^2^ The endoscopic placement of a posterior cartilage graft pioneered by Inglis, is well‐established and provides patients with laryngeal expansion without the need for an incision of the neck or in the anterior laryngeal framework.^3^ We aimed to validate in an animal model the feasibility of a novel technique of endoscopic anterior cricoid split (ACS) and rib grafting. The goal is to place and secure an anterior subglottic graft without opening the neck. We hereby describe our surgical technique and the challenges encountered.

## Methods

### Ethical Considerations

The procedures were conducted at an approved animal surgical laboratory at King Saud university medical city and King Faisal specialist hospital and research center. The protocol employed was ethically reviewed and approved by the animal care and use committee, King Faisal specialist hospital and research center (ID: RAC# 2222001).

### Study Design, Sample Size, and Inclusion Criteria

This was a pilot feasibility study performed on 3 Ardhi goats aged 3 months, with an average weight of 15 kg.^4^ We confirmed the presence of normal laryngotracheal anatomy prior to inclusion in the study.

### Outcome Measures

The primary outcome measure was the feasibility of the surgical technique. The secondary outcome measures were the stability of the cartilage graft, the status of the graft healing, and airway patency on surveillance bronchoscopies 7 days postoperatively.

### Anesthesia Protocol

The animal was premedicated with intramuscular injection (IM) of midazolam 0.2 mg/kg. Then, general anesthesia induction with midazolam 0.1 mg/kg IM and 10 mg/kg of ketamine IM. Inhalant anesthetic was administered through insufflation port of the laryngoscope with 5% isoflurane during induction and 2% to 3% isoflurane for anesthesia maintenance.

Prophylactic antibiotic was administered following induction in the form of intramuscular injection of 1.5 mL (150 mg/mL) of amoxicillin corresponding to 7 mg/kg. At the start of bronchoscopy, a single intramuscular injection of 1 mL (20 mg/mL) of dexamethasone was administered followed by daily dose of 0.7 mg/kg/d for 3 days. Intravenous injection of Flunixin 1.1 mg/kg q12 hours was given for pain control.

### Experimental Procedure Technique

The technique was developed by author A.A. The larynx was exposed using a size JV laryngoscope (Karl Storz GmbH & Co KG) and examined using a 4‐mm, 0‐degree Hopkins rod‐lens telescope (Karl Storz GmbH & Co KG). Topical anesthesia of the larynx was introduced with 10% lidocaine spray. After confirmation of a normal airway anatomy, the laryngoscope was removed.

**Figure 1:**
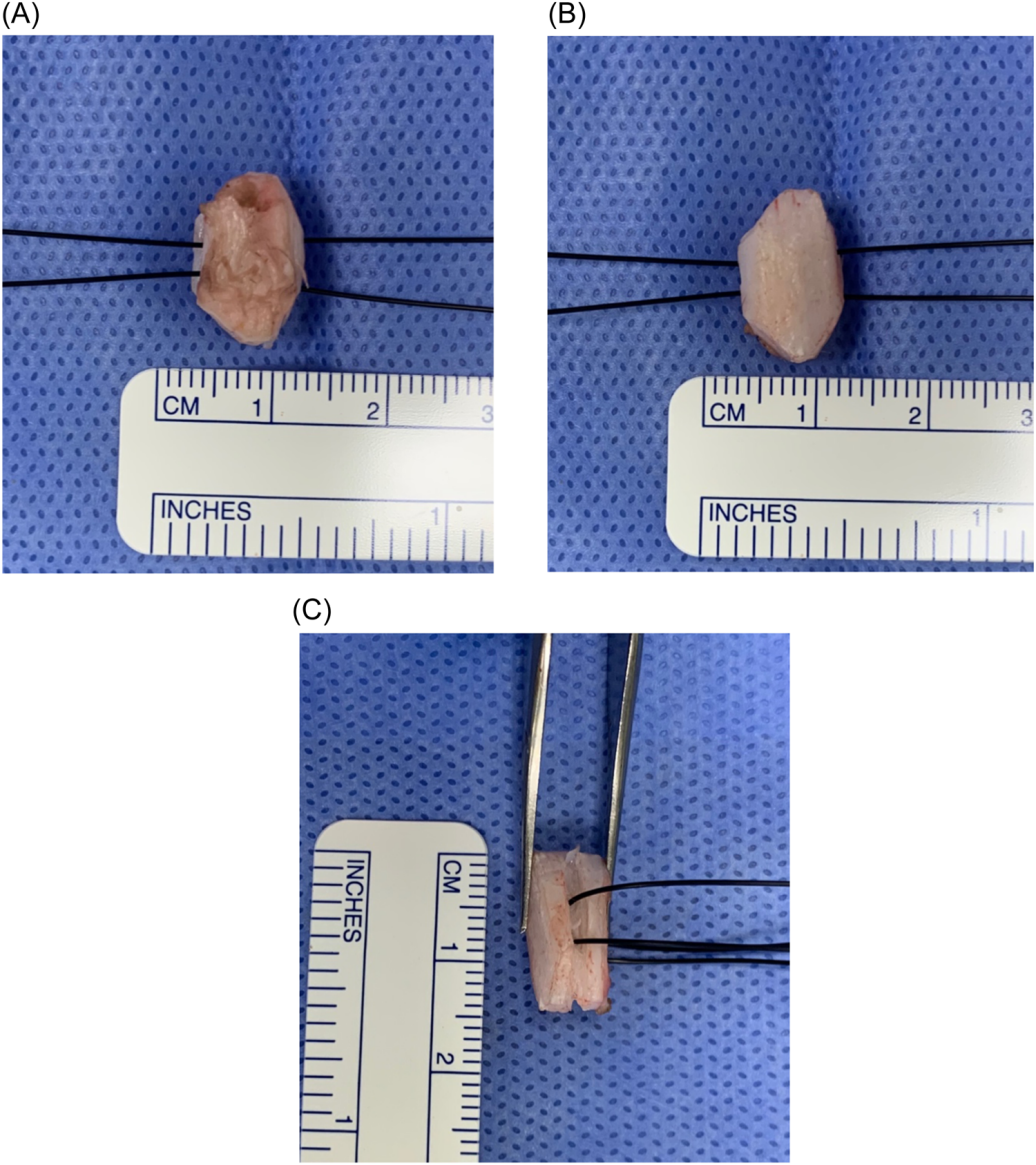
Cartilage graft shape. (A) Anteroposterior view showing the surface with intact perichondrium. (B) Anteroposterior view showing the surface without perichondrium. (C) Side view showing the entry points of the sutures.

Rib graft harvest was carried out from the right 5th rib. Then, the graft was shaped into a hexagone shape with right and left trapezoid side grooves. The side grooves were designed to be closer to the surface which had an intact perichondrium. Two separate ETHILON size 1 (Johnson & Johnson intl) sutures were passed through the side grooves perpendicular to the long axis of the graft (Figure 1A‐C).

**Figure 2:**
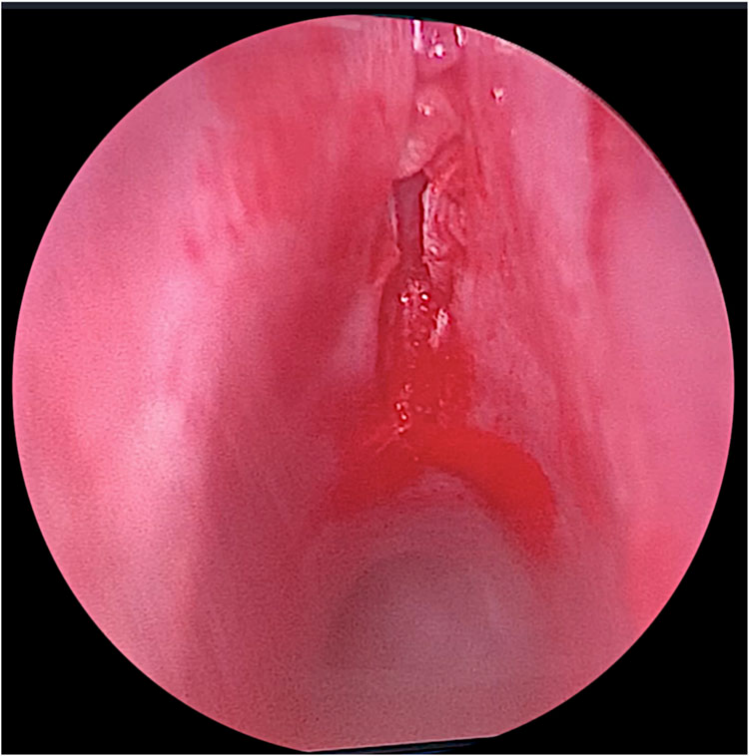
Endoscopic midline anterior split of the cricoid cartilage and first tracheal ring. Endoscopic laryngeal view showing the anterior midline incision of the cricoid cartilage and first tracheal ring.

**Figure 3:**
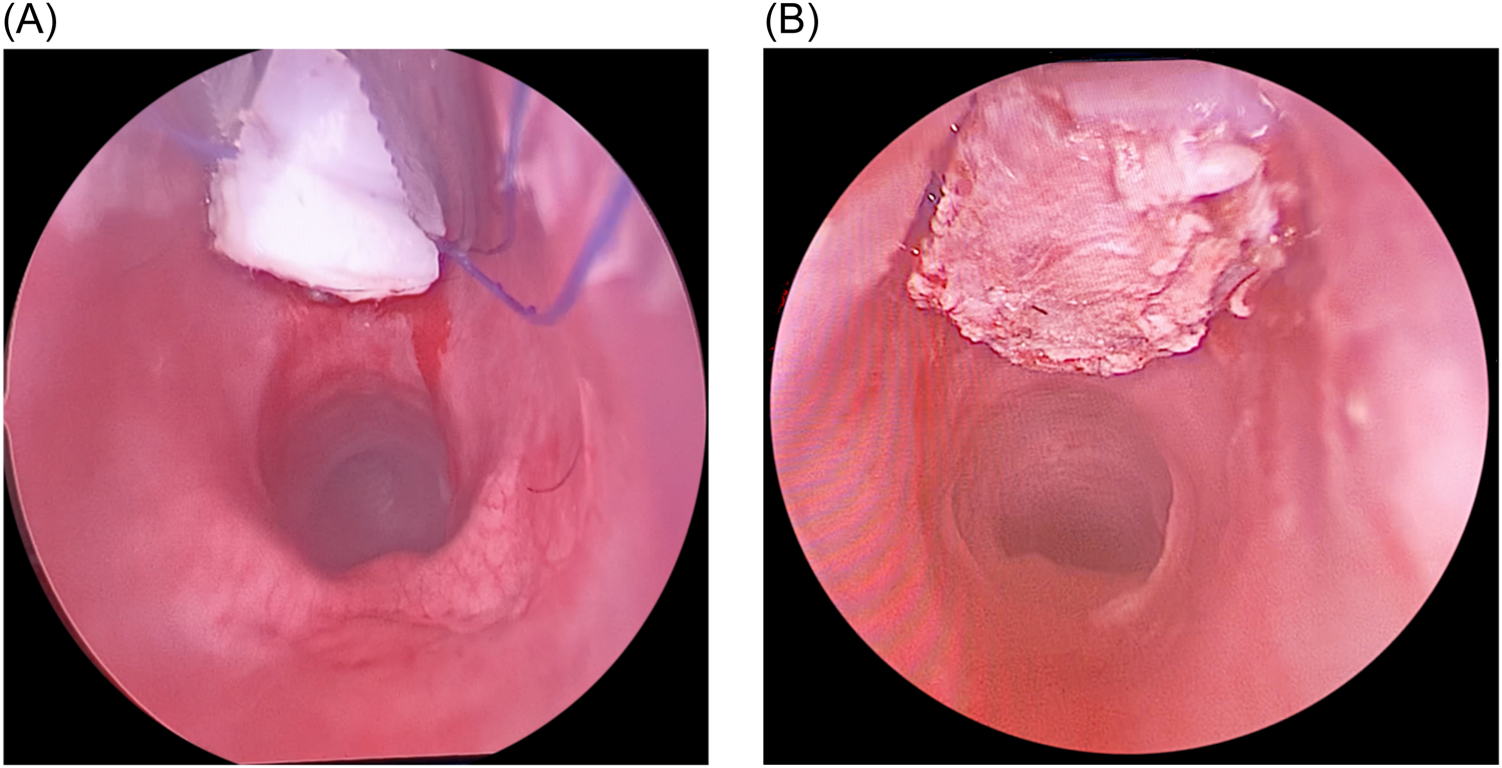
Laryngoscopic view of the anterior graft. (A) Endoscopic laryngeal view during graft placement. (B) Endoscopic laryngeal view showing the final position of the cartilage graft.

**Figure 4:**
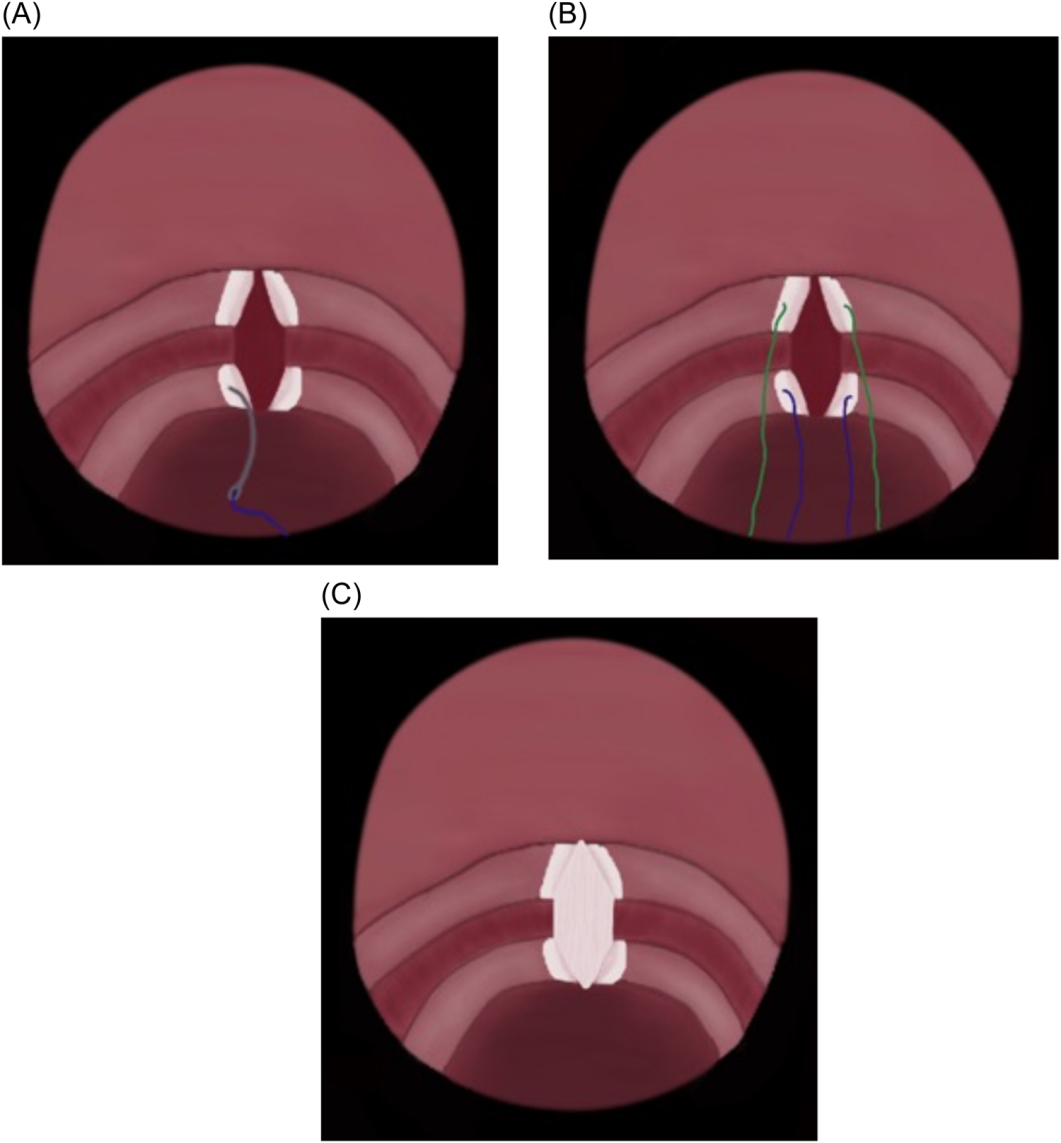
Schematic representation of the surgical technique. (A) The first graft stabilizing suture passing through the divided first tracheal cartilage. (B) Placement of the 4 graft stabilizing sutures. (C) The final position of the cartilage graft.

The laryngoscope was then placed back and an endoscopic midline anterior cricoid split was performed extending through the first tracheal ring using a round knife (Figure 2). The area was then palpated with an alligator forceps to ensure that the incision was involving the full thickness of the cartilage. Subsequently, a balloon dilation was performed to allow expansion of the divided cartilaginous rings. A laryngeal needle holder was utilized to pass the needle on 1 end of the lower graft suture, into the divided 1st tracheal cartilage, 5 mm away from its medial edge until it was picked up externally from the neck skin. Then, the other end is threaded through a half‐circle, cutting, free Mayo needle and the needle was passed 5 mm away from the medial edge of the cartilage on the other side. The process is sequentially repeated for the upper graft suture through the divided cricoid cartilage. The assistant gradually pulled the 2 lower suture ends then the 2 upper ends from the neck, while at the same time, the main surgeon carefully positions the graft into the anterior split (Figures 3 and 4A‐C). After confirmation of appropriate graft position via telescopic examination, the sutures are tied on a silastic sheet over the anterior neck skin (the right upper and lower threads were tied together then the left upper and lower threads were tied together to obtain maximum suspension of the graft). Postoperatively, daily assessment of the breathing pattern was performed, body temperature was taken, and the neck was examined for the possibility of surgical emphysema.

The surgical technique was modified in the last animal to include a laryngeal stent which was designed from an endotracheal tube size 7 that was cut into a 3‐cm‐long stent. Following subglottic graft positioning and suspension, the laryngeal stent was placed endoscopically and suspended with endo‐extra laryngeal sutures utilizing 1.O Prolene suture (video).

## Results

**Figure 5:**
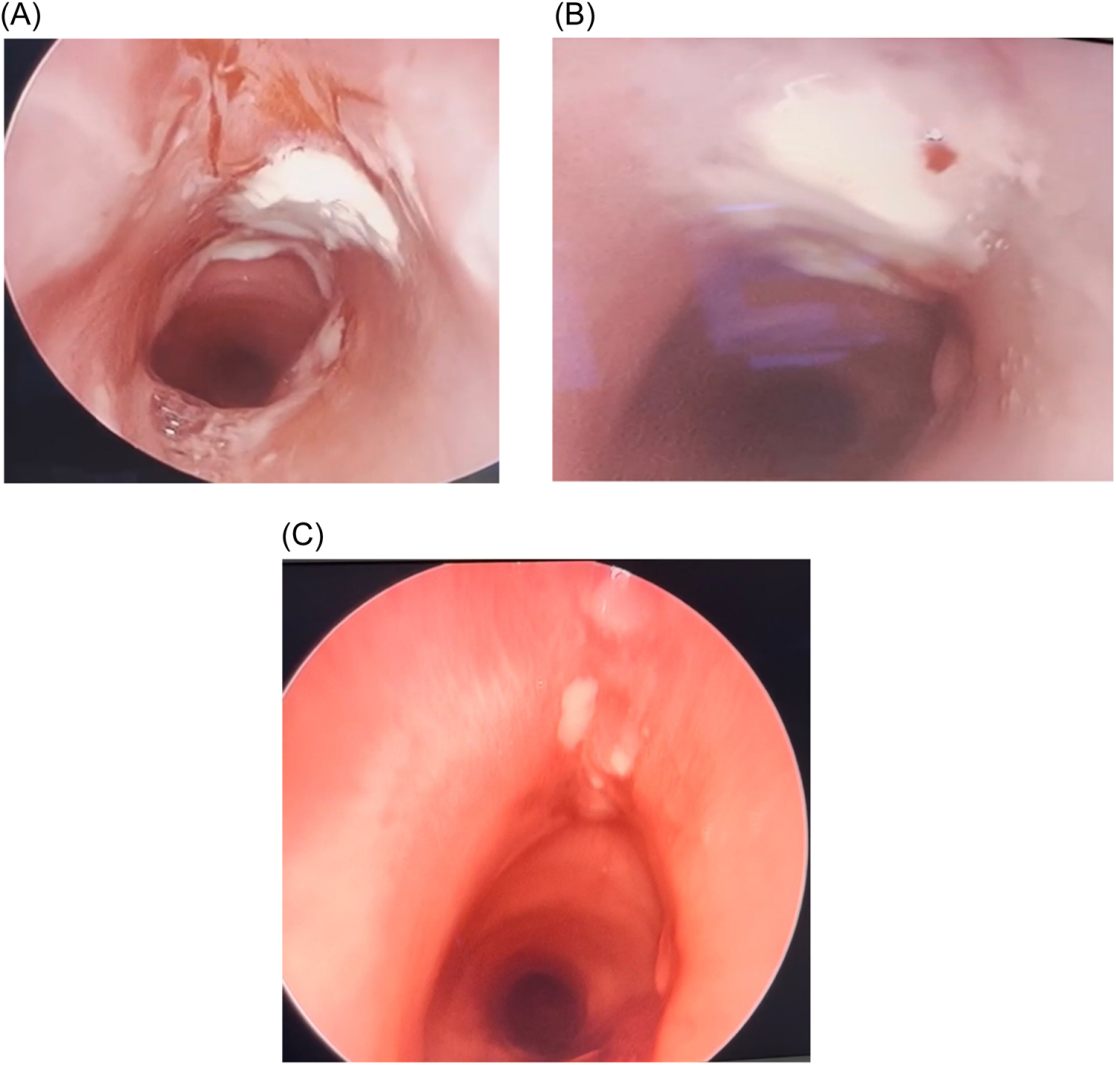
Follow up direct laryngobronchoscopy examinations post stent removal. (A) Follow up at 2 weeks. (B) Follow up at 3 weeks. (C) Follow up at 4 weeks.

The surgery was achieved successfully in all 3 animals, with no intra‐operative complications. Postoperatively, the animals were breathing comfortably without any signs of airway distress. There was no evidence of surgical emphysema in the neck or the chest, and no fever. Once daily cefazolin injection (40 mg/kg/d) was administered for 7 days. Surveillance bronchoscopy performed on postoperative Day 7 revealed that the subglottic airway was patent and the graft was in‐place without evidence of graft prolapse. However, the first goat developed evidence of surgical site infection within the larynx leading to partial graft resorption. In the third goat, the laryngeal stent was removed after 2 weeks during surveillance DLB. Then, the graft‐suspension sutures and silastic sheet were removed on postoperative Day 21. DLB examination on postoperative Day 30 revealed that the subglottic airway remained patent and the graft was in‐place with full mucosa coverage (Figure 5A‐C).

## Discussion

Open ACS was introduced in 1980 for premature infants with multiple failed extubation attempts.^5^, ^6^ The endoscopic approach of ACS enabled performing this technique without the need for a neck incision, thus resulting in less patients' morbidity.^6^ The added use of balloon dilation (BD) following ACS was reported to be a treatment option for infants with failure of extubation as well as pediatric SGS.^6^, ^7^

Laryngotracheal reconstruction (LTR) with anterior and/or posterior graft is an effective procedure for expanding the stenotic airway in both children and adults.^8^, ^9^ The reported success rate in achieving decannulation following LTR is 89% to 97%, 78% to 91%, and 50% to 72% for SGS grades II, III, and IV, respectively.^9^ Alternatively, surgical outcomes following LTR in nontracheostomized patients can be expressed by the recurrence of symptoms and the need for additional surgeries. When LTR is performed as a single stage, tracheostomy and its complications can be avoided.^8^ However, double‐stage LTR is a safer alternative in the presence of poor pulmonary reserve, comorbidities, or in case of multilevel stenosis.^8^

The use of domestic mammals' models in endoscopic and open airway surgeries has been described as an accessible and, to some extent, a comparable model to human anatomy.^10^, ^11^, ^12^ In this animal model we performed the procedure on goat as it had the required airway diameter to accommodate the surgical instruments available in our animal laboratory. We demonstrated the feasibility of this novel procedure which is potentially useful in patients who are candidates for single‐stage reconstruction. In humans, performing a single‐stage laryngeal reconstruction necessitate a period of endotracheal intubation to support the graft in place. Thus, in the last animal we utilized an endotracheal tube as a stent to support the graft until adequate initial healing was ensured. However, in humans we recommend to initially perform this procedure as a double stage in patients who already have a tracheostomy in place. Also, the needle size that was utilized in the animal model (ETHILON size 1) is large in comparison to the airway caliper in small children and surgeons are advised to tailor the needle size choice to the patient's airway size. To the best of our knowledge, this technique has not been described in the literature. Future studies should validate the use of this technique and its effectiveness in a model with subglottic stenosis.

The main limitation was the inability to keep the animal intubated, ventilated, and monitored for a period of time in our animal laboratory to avoid the use of stent as in human due to the limited resources within the center which could have led, in the authors opinion, to the infection in the first animal.

## Conclusion

In conclusion, endoscopic ACS and rib grafting is a feasible procedure that can be potentially useful for patients who are candidates for a double‐stage reconstruction of the subglottis. Future studies should investigate the safety and validity of this technique in a model with subglottic stenosis.

## Author Contributions

**Bshair Aldriweesh**, study design, coordinator, applying the surgical technique, data acquisition, and drafting of the manuscript; **Nasser Almutairi**, applying the surgical technique, data acquisition, and surgical video production; **Waleed Alshareef**, Applying the surgical technique and data acquisition; **Abdullah Sindi**, applying the surgical technique; **Ahmed Alammar**, study conception and design and applying the surgical technique.

## Disclosure

### Competing interests

None.

### Funding source

None.

## Supporting information

Supporting information.

## Data Availability

All data analyzed during this study is included in the published article.
